# Network Pharmacology and Molecular Docking Explore the Mechanism of Mubiezi-Yinyanghuo Herb Pair in the Treatment of Rheumatoid Arthritis

**DOI:** 10.1155/2023/4502994

**Published:** 2023-12-08

**Authors:** Fuxue Meng, Xiaomai Tao, Longkuan Li, Wei Jia, Xin Yang, Yuchen Yang

**Affiliations:** ^1^The Third Affiliated Hospital of Guizhou Medical University, Duyun, Guizhou, China; ^2^Guizhou Medical University, Guiyang, Guizhou, China

## Abstract

**Objective:**

Our previous studies have shown that the Mubiezi-Yinyanghuo (MBZ-YYH) herb pair inhibits rheumatoid arthritis (RA) cell proliferation and glycolysis, promising results with an obscure mechanism of action.

**Methods:**

Therefore, it is necessary to explore the main components of MBZ-YYH and unravel the potential mechanism in RA based on network pharmacology and molecular docking methods. Components and targets of MBZ-YYH were retrieved from the TCMSP. Relevant targets of RA were searched in GeneCards, therapeutic target database (TTD), and DisGeNET databases; the common targets of the MBZ-YYH compounds and RA were obtained by comparison; and a component-target interaction network was established by Cytoscape 3.9.1. Gene ontology (GO) analysis and Kyoto Encyclopedia of Gene and Genome (KEGG) pathway enrichment analysis were performed through the David database. Molecular docking was performed by PyMoL2.3.0 and AutoDock Vina1.1.2 software.

**Results:**

7 active ingredients and 58 putatively identified target genes were screened from MBZ, and 16 effective components of YYH and 230 potential targets were identified. There were 29 mutual targets between the two herbs and RA. Through the PPI network, 9 hub targets which contain JUN, CASP3, PPARG, PTGS2, GSK3B, CASP8, HMOX1, ICAM1, and HK2 were screened out. GO term enrichment analysis indicated that positive regulation of the apoptotic process, response to drugs, and response to hypoxia were significantly enriched. Based on KEGG analysis, it was mainly associated with the IL-17 signaling pathway, the TNF signaling pathway, and the p53 signaling pathway. The docking analysis revealed that the effective components showed strong binding activity with the receptors.

**Conclusion:**

The effects of the MBZ-YYH herb pair on RA were coordinated by the interaction of diverse components, which may be through the IL-17 signaling pathway and the TNF signaling pathway, which target GSK3B, HK2, caspase 3, and caspase 8, inhibiting the proliferation and glycolysis of rheumatoid arthritis fibroblast-like synovial cells (RA-FLS) and tending towards an increasing efficacy and decreasing toxicity effect on RA.

## 1. Introduction

Rheumatoid arthritis (RA) is a chronic, inflammatory, and autoimmune disease characterized by inflammatory changes of synovial tissue, cartilage, and joint skeleton [1]. The common clinical manifestations of RA include joint swelling, stiffness, and dysfunction [2]. This eventually leads to disability and ultimately death [3, 4]. RA can affect people of all ages; around 1% of them suffer from intractable pain. It not only causes great suffering for patients and family members but also brings heavy financial burden for individuals and society [5]. Drugs that reduce joint pain or retard the progression of disease include nonsteroidal anti-inflammatory drugs (NSAIDs), disease-modifying antirheumatic drugs (DMARDs), and steroids and biological response modifiers [6]. However, these drugs can produce serious side effects, including renal toxicity, gastrointestinal toxicity, hepatic damage, and myelosuppression [7–10]. Moreover, it has been associated with an increased prevalence and incidence of cardiovascular diseases [11, 12].

**Figure 1:**
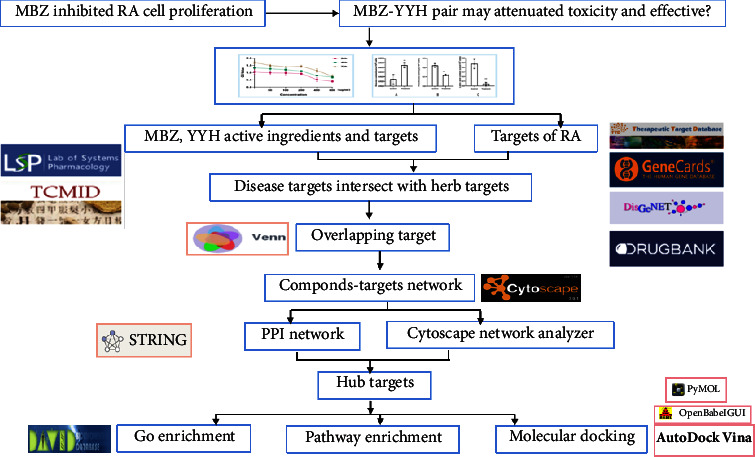
Flowchart of network pharmacology of MBZ-YYH in the treatment of RA.

With developments in the field of traditional Chinese medicine (TCM), the treatment of RA has entered a stage of diversified comprehensive treatment. As an assistant and substitute medicine, TCM has gained increasing attention because of significant efficacy and lesser side effects. Our previous studies found that an extract of MBZ could suppress the angiogenesis of human umbilical vein endothelial cells (HUVEC) in the RA synovial cavity by inhibiting glycolysis, but the specific mechanism is still unclear [13]. MBZ has the definite effects of dispersing swelling and healing sores. Pharmacological research has revealed that in addition to anticancer, anti-inflammation, and antibacterial pharmacological effects based on the traditional efficacy, MBZ also exhibits antiulcer, antioxidation, and immune regulation effects [14]. MBZ is an effective but toxic traditional Chinese medicine; therefore, the present study was carried out to search for compatible herbs that can enhance the efficacy and reduce the toxicity in treatment of RA. Kan et al. [15] showed in an interrelated study that the optimal ratio 1 : 1 of MBZ with YYH combined showed more prominent antitumor proliferation; moreover, there was no conspicuous effect on the growth of LO2 normal human liver cells, and it was speculated that the compatibility of the two herbs has the synergistic effect of enhancing efficacy and reducing toxicity. Considering this, current research has bestowed the MBZ-YYH pair on HFLS-RA to detect its effects on cell proliferation and glycolysis. Through network pharmacology and molecular docking, we explored the action mechanism of MBZ on RA under the coordination of YYH, in expectation of providing a theoretical basis for the extensive clinical treatment of RA. The flowchart of this study is presented in Figure 1.

## 2. Materials and Methods

### 2.1. Materials

Human rheumatoid arthritis synovial fibroblasts (HFLS-RA) were purchased from Beina Chuanglian Biotechnology Co., Ltd.; fetal bovine serum (Sciencell); RPMI 1640 medium (Gibco); cell counting kit-8 (Beijing Solarbio); glucose content detection kit, ATP content detection kit, lactic acid content detection kit, and hexokinase activity detection kit (Beijing Solarbio).

### 2.2. Methods

#### 2.2.1. Cell Viability Was Detected by the Cell Counting Kit-8 (CCK-8)

HFLS-RA in the logarithmic growth phase was collected by centrifugation, counted, and diluted to 1 × 10^4^/mL in Dulbecco's modification of Eagle's medium with 10% fetal bovine serum, and 100 *μ*L per well was added to a 96-well plate and adherent cultured overnight. The mixture of MBZ and YYH (1 : 1, by weight, refers to Kan's study [15]) was diluted in DMEM medium (concentration gradient was set as 0, 50, 100, 200, 400, and 800 *μ*g/mL) and cultured for 24, 48, and 72 hours. CCK-8 (10 *μ*L) was added, and the absorbance was detected at 490 nm by a microplate reader.

#### 2.2.2. Determination of Glucose (Glu) Content, Hexokinase (HK) Activity, and Lactate (LA) Content

Cell glucose content was measured by spectrophotometry according to the manufacturer's instructions. G (*μ*mol/10^4^ cells) = 0.002 × (measuring tube−blank tube)/(standard tube−blank tube); HK (U/10^4^ cells) = 2.226 × ΔA; and LA (*μ*mol/10^6^ cells) = 0.2375 × X.

#### 2.2.3. Establishment of Active Ingredients and RA Disease Target Databases of MBZ and YYH

By searching for a traditional Chinese medicine (TCM) system pharmacology (TCMSP) platform (http://tcMBZpw.com/tcMBZp.php), the processes that determine the pharmacokinetic behavior of a drug compound are its absorption, distribution in the body, metabolism, and elimination from the body (ADME). The specific screening criteria were oral bioavailability (OB) ≥30% and drug‐like properties (DL) ≥0.18. “Rheumatoid arthritis” as keywords retrieval in the GeneCards database (https://www.genecards.org/), Therapeutic Target Database (TTD) (http://db.idrbla.net/ttd/), and DisGeNET(http://www.disgenet.org/) databases to obtain RA-related disease targets. The intersection between the compounds and RA disease targets was obtained by the Venn diagram.

#### 2.2.4. Protein-Protein Interaction (PPI) Network of Potential Targets of the MBZ-YYH Herb Pair and RA

The intersection of targets obtained by Venn diagram was regarded as the potential targets of compounds for the treatment of RA. The intersection of the other targets of MBZ and YYH was regarded as the interactional targets, and the protein-protein interaction (PPI) network of these potential targets was constructed by STRING (https://string-db.org/cgi/input.pl). Cytoscape 3.9.1 (https://cytoscape.org/) was used for visualization, and the hub targets were screened according to the degree value.

#### 2.2.5. GO and KEGG Pathway Enrichment Analysis

By the DAVID database (https://david.ncifcrf.gov/), the *P* value less than 0.05 was set as a condition of screening for enrichment of gene ontology (GO) function analysis and Kyoto Encyclopedia of Genes and Genomes (KEGG) pathway enrichment analysis of the key target protein. Three modules, namely, biological process, cellular component, and molecular function, were selected for mapping. Its first 10 entries were visualized as bubble plots using the ImageGP (https://www.bic.ac.cn/ImageGP/).

#### 2.2.6. Molecular Docking

Referring to studies in [16–18], the nine highest-degree targets in the “target-compound” network were regarded as receptors, and core compounds and anti-RA drugs were regarded as ligands. The crystal structures of the 9 proteins were downloaded from the Protein Data Bank (http://www.rcsb.org/pdb) and saved in PDB format. Three-dimensional (3D) conformation of candidate compounds (https://pubchem.ncbi.nlm.nih.gov/) was downloaded from the PubChem database and saved in SDF format and then converted into a PDB format via Open Babel. Ligands and receptors were prepared by AutoDock Vina and PyMOL (v.2.3.0). The original ligand and water molecules were removed from the crystal structure of the receptor, nonpolar hydrogen was added, and the Gasteiger partial charge was calculated. Applying energy minimization and assigning atomic charges to process ligands, the active site of molecular docking is determined by the ligand coordinates in the target protein complex. The ligand is set to be flexible while the receptor is rigid. All prepared receptors and ligands were saved in pdbqt format. The affinity of the binding strength between the ligand and the target protein was assessed with AutoDock, and the conformation with the best affinity was selected as the final docking conformation and visualized in Pymol 2.3.0. The stability of systems was evaluated using the root-mean-square deviation (RMSD) of the aligned protein-ligand coordinate set calculated against the initial frame.

## 3. Results

### 3.1. MBZ-YYH Herb Pair Inhibited the Proliferation of RA Synovial Fibroblast Cells

**Figure 2:**
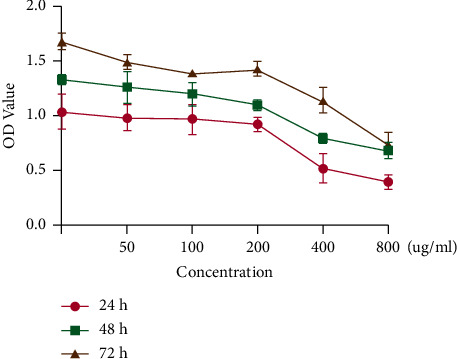
CCK-8 detection of the effect of MBZ and YYH on RA cells. The concentration is greater than 200 *μ*g/ml; the difference is significant. 200 *μ*g/ml (*P*=0.029), 400 *μ*g/ml (*P*=0.0008), and 800 *μ*g/ml (*P*=0.002).

To determine the effect of the MBZ-YYH herb pair on RA cells, we treated RA cells with a 1 : 1 ratio of MBZ and YYH in a concentration gradient and a time gradient. The results showed that the proliferation of RA cells was significantly inhibited by 400 *μ*g/ml of the MBZ-YYH herb pair at 24 h (*P* < 0.001) (Figure 2), so we took this concentration as the best concentration for subsequent experiments.

### 3.2. Inhibition of RA Cell Glycolysis by the MBZ-YYH Herb Pair

**Figure 3:**
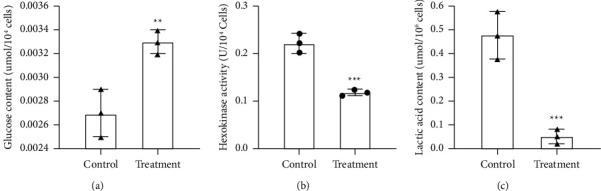
Effects of MBZ and YYH on glycolysis of RA cells. (a) Determination of glucose content (^*∗∗*^*P*=0.0097); (b) hexokinase activity assay (^*∗∗∗*^*P*=0.001); and (c) determination of lactic acid content (^*∗∗*^*P*=0.002). Here, control means no drug. Treatment shows the cells treated with MBZ and YYH.

To further explore the effect of the MBZ-YYH herb pair on the glycolysis of RA cells, glucose and lactate contents and hexokinase activity were measured. The results showed that the contents of glucose (^*∗∗*^*P* < 0.01) and lactic acid (^*∗∗*^*P* < 0.01) and the activity of hexokinase (^*∗∗∗*^*P* ≤ 0.001) in RA cells were recovered by the combination of MBZ and YYH, prompting that the MBZ-YYH herb pair could inhibit the glycolysis of RA cells (Figure 3).

### 3.3. Chemical and Pharmacological Analysis for Active Ingredient of the MBZ-YYH Herb Pair

From the TCMSP database, a total of 31 constituents of MBZ and 130 compositions of YYH were retrieved. Through the analysis of physical and chemical properties from TCMSP, it was found that the molecular weight (MW), octanol-water partition coefficient (AlogP), and hydrogen acceptor (Hacc) were prominently different between the two herbs (*P* < 0.05), while the hydrogen donor (Hdon), OB, and DL were not different. Indicating that the chemical properties of the two species were obviously different, however, to some extent, there is a certain intersection between them. Therefore, it is speculated that some components of this compatibility may be mutually beneficial, while others are mutual detoxication (Table 1).

According to the threshold requirements of OB ≥ 30% and DL ≥ 0.18, the active ingredients of MBZ and YYH were screened. Considering that saponins are the main components of MBZ, oleanolic acid (OB = 29.02) was retained for target analysis to ensure accuracy. In addition, the OB values of NON (OB = 26.74%, DL < 0.04), karounidiol (OB = 26.26%, ODL = 0.77), and MOL012372 (OB = 28.3%, DL = 0.77) were similar to the mean value of the OB components of MBZ, so they were selected as candidate ingredients for further analysis (Table 2).

### 3.4. Network Construction of the Effective Components Corresponding Targets of the MBZ-YYH Herb Pair for RA Therapeutic Targets

**Figure 4:**
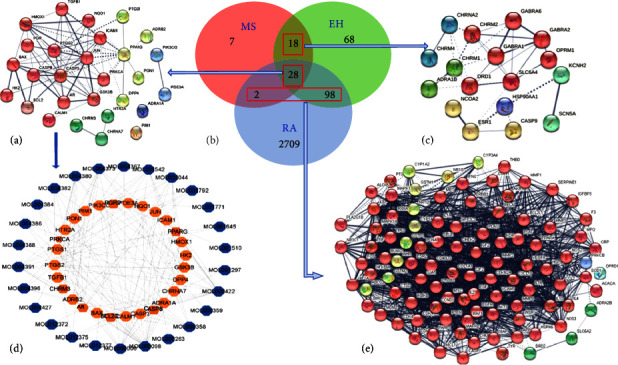
Network of compound targets and RA targets. (a) MBZ-RA-YYH PPI network; (b) Venn diagram showed that RA and compound targets had 28 common targets; (c) MBZ-YYH PPI network; (d) potential target network diagram; and (e) MBZ-RA/YYH-RA PPI network.

There were 55 and 212 targets corresponding to the active ingredients of MBZ and YYH, respectively. To further understand the role of targets, we obtained the target intersection that was regarded as a potential therapeutic target through the Venn diagram and constructed a PPI network. The resulting PPI network was introduced into Cytoscape 3.9.1, and hub targets were selected according to the degree values which produced form network analyzer of Cytoscape (Figure 4). Aggregately, 28 targets of the two compounds intersected with RA targets (it should be noted that HK2 was included in the target set of MBZ as evidence from previous studies). Altogether 9 targets, which included the top 8 with the highest degree value with the addition of HK2 (Table 3), were used as receptors for molecular docking, and the corresponding compounds of potential targets (6 of MBZ and 12 of YYH) were used as ligands.

### 3.5. GO and KEGG Enrichment Analysis

**Figure 5:**
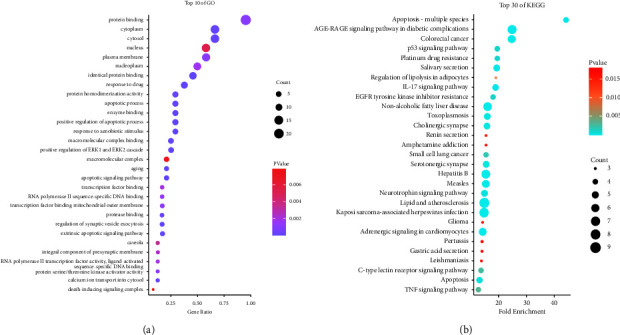
GO and KEGG enrichment analyses of potential targets. (a) GO enrichment analysis; (b) KEGG enrichment analysis.

For the purpose of comprehending the molecular functions of potential targets, GO and KEGG enrichment analyses were performed. The results showed that GO was mainly involved in protein binding, positive regulation of the apoptotic process, response to drugs, and response to hypoxia. KEGG mainly refers to EGFR tyrosine kinase inhibitor resistance, the IL-17 signaling pathway, the TNF signaling pathway, and the p53 signaling pathway (Figures 5(a) and 5(b)). It is hinted that MBZ and YYH may regulate the response of cells to drugs and the hypoxia environment through the IL-17 signaling pathway and the TNF signaling pathway.

**Figure 6:**
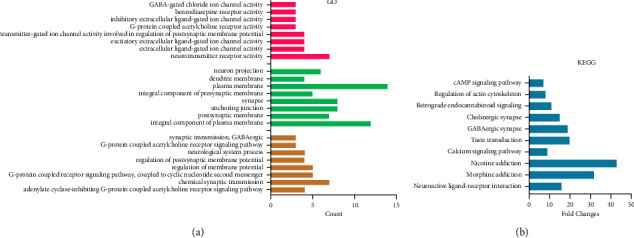
GO and KEGG enrichment analyses of common targets in MBZ-YYH. (a) GO enrichment analysis. (b) KEGG enrichment analysis.

In addition, we also conducted GO and KEGG analyses on 18 common targets of MBZ-YYH, and the results showed that it mainly involves G-protein-coupled receptor activity, extracellular ligand-gated ion channel activity, regulation of membrane and neuroactive ligand-receptor interaction, calcium signaling pathway, and regulation of actin cytoskeleton (Figures 6(a) and 6(b)). It is allusived that MBZ-YYH interaction may lead to extracellular ligand-receptor interaction through the activation of calcium ion channels by G-protein-coupled receptors on the membrane.

### 3.6. Molecular Docking

**Figure 7:**
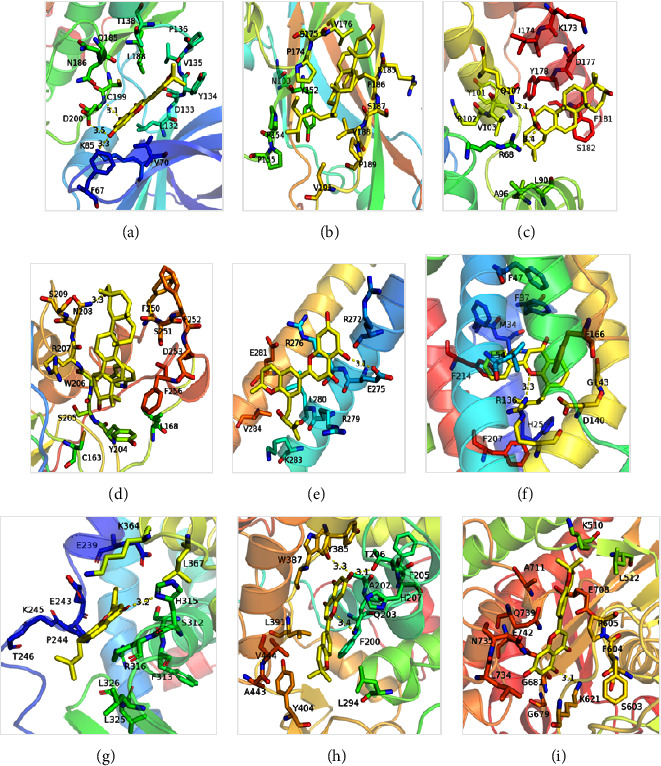
Molecular docking visualization. (a) GSK3B-Yinyanghuo C (−9.4 kcal/mol), (b) ICAM1-bessisterol (−8.0 kcal/mol), (c) CASP8-oleanolic acid (−8.9 kcal/mol), (d) CASP3-3R,4aR,6aS,6bS,8aS,11R,12aR,14bS)-4,4,6a,6b,8a,11,14b-heptamethyl-11-methylol-1,2,3,4a,7,8,9,10,12,12a,13,14-dodecahydropicen-3-ol (−8.8 kcal/mol), (e) JUN-Yinyanghuo A (−7.3 kcal/mol), (f) HMOX1-8-(3-methylbut-2-enyl)-2-phenyl-chromone (−9.3 kcal/mol), (g) PPARG-8-(3-methylbut-2-enyl)-2-phenyl-chromone (−9.7 kcal/mol), (h) PTGS2-Yinyanghuo C (−10.7 kcal/mol), and (i) HK2-Yinyanghuo C (−9.6 kcal/mol).

18 active ingredients (MBZ : 6; YYH : 12) and 9 interest proteins with the highest degree values (HK2, 3) for molecular docking. The results showed that the binding energy of all proteins to the active components is less than −5 kcal/mol, indicating that ligands and receptors were closely bound. The strongest proteins that bind to the active ingredients are as follows: PPARG and 8-(3-methylbut-2-enyl)-2-phenyl-chromone (−9.7 kcal/mol), PTGS2-yinyanghuo C (−10.7 kcal/mol), HK2-Yinyanghuo C (−9.6 kcal/mol), (CASP3-3R,4aR,6aS,6bS,8aS,11R,12aR,14bS)-4,4,6a,6b,8a,11,14b-heptamethyl-11-methylol-1,2,3,4a,7,8,9,10,12,12a,13,14-dodecahydropicen-3-ol (−8.8 kcal/mol), JUN-Yinyanghuo A (−7.3 kcal/mol), HMOX1- 8-(3-methylbut-2-enyl)-2-phenyl-chromone (−9.3 kcal/mol), GSK3B-Yinyanghuo C (−9.4 kcal/mol), ICAM1-bessisterol (−8.0 kcal/mol), and CASP8-oleanolic acid (−8.9 kcal/mol) (Figure 7).

**Figure 8:**
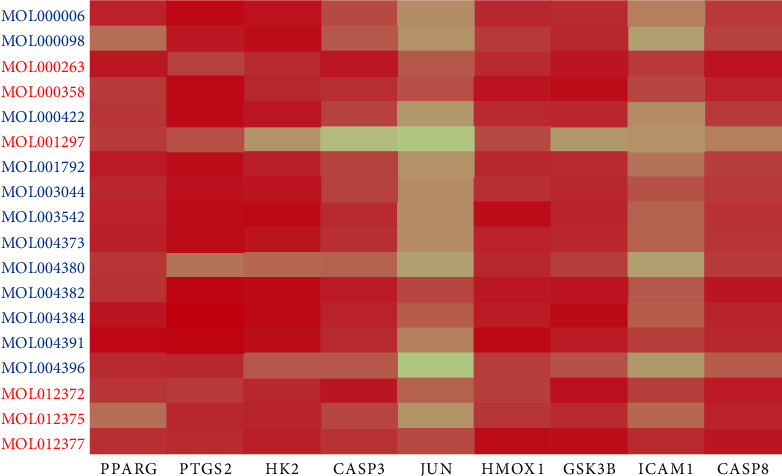
Binding energy heat map of protein and active ingredient molecule docking. The active ingredient is shown vertically; the active ingredient of MBZ is shown in red; and YYH is shown in blue. The protein is shown horizontally.

Except ICAM1-bessisterol, which only has hydrophobic interaction, the other eight pairs all interact through hydrogen bond formation and hydrophobic force. Among them, PTGS2-Yinyanghuo C (−10.7 kcal/mol) is well paired, and Yinyanghuo C is most likely to combine with HK2 and GSK3B at the same time. It is worth noting that the effect of MBZ on HK2 is mainly achieved by MOL012372, MOL012375, MOL012377, MOL000263, and MOL000358 (all ≤−8 kcal/mol); YYH mainly acts on HK2 through MOL003542, MOL004382, and MOL004384 (all ≤−9.5 kcal/mol) (Figure 8).

## 4. Discussion

In this study, it was found that the MBZ-YYH herb pair can inhibit proliferation and activity of hexose kinase of HFLS-RA. Further, it can restore the glucose level and reduce the content of lactic acid, suggesting that the MBZ-YYH herb pair may inhibit the glycolysis of RA cells. This finding is consistent with our previous study that MBZ treated RA by glycolysis inhibition [13]. Nevertheless, the mechanism of the active ingredients is still uncertain. Therefore, network pharmacology and molecular docking were used to explore the underlying mechanism.

Chemistry and pharmacology analysis shows that there are obvious differences in the chemical properties between MBZ and YYH, but they overlap to some extent. It is hinted that there may be some components that mutual promotion and mutual-assistance, mutual restrain and mutual-detoxication. Herbs interaction is reflected in the fact that each component can not only act independently but also generate new modes and mechanism of action between the components after their compatibility [19]. Among the compatibility interaction mechanisms involving direct and indirect effects of components, this study may be more inclined to the latter, namely, components affect the absorption and metabolism of different components by acting on certain targets in the organism. The results can be manifested as influencing the effect of each other to achieve the desired treatment effect.

Network pharmacology analysis suggested that the common hub targets of the main components of herbs and RA were JUN, CASP3, PPARG, PTGS2, GSK3B, CASP8, HMOX1, ICAM1, and HK2. GO and KEGG analyses showed that the main targets involved response to drugs, response to hypoxia, IL-17 signaling pathway, and TNF signaling pathway, suggesting that the treatment of RA by the MBZ-YYH herb pair may regulate the response of cells to drugs and the hypoxia environment through the IL-17 signaling pathway and TNF signaling pathway.

During the development of RA, hypoxia alters cell biological functions by inducing mitochondrial dysfunction and promoting the switching of glycolytic pathways, leading to abnormal cell invasion and angiogenesis [20]. Our previous study showed that the extract of MBZ regulated angiogenesis in RA through glycolysis inhibition, which was similar to this result [13]. Although some neovascularization provides oxygen to the increased inflammatory cell mass, the neovascularization network is dysfunctional and unable to restore tissue oxygen homeostasis, keeping RA joints in a hypoxic environment [21]. Due to the high metabolic demand of synovial cells, the lactate content in the synovial fluid of RA is significantly increased and the glucose concentration is significantly decreased [22–24]. The results of the present study are in accordance with the findings of the abovementioned, as increased glycolytic enzyme activity in RA synovial tissue, RA synovial cell proliferation, and massive inflammatory cell infiltration lead to increased local capillary oxygen diffusion distance and oxygen consumption, resulting in RA joint cavity hypoxia [25, 26]. Hypoxia-inducible factor 1(HIF-1), a nuclear transcription factor that regulates cellular oxygen homeostasis and the expression of hypoxia-responsive genes, is increased under hypoxic conditions. The gene expression of sugar transport and glycolytic enzymes was increased to promote glycolysis to adapt to the energy deficiency of cells under hypoxia [27]. Cell proliferation and activation in the inflammatory joint cavity require the conversion of glucose metabolism to a high-energy state of glycolysis to maintain energy homeostasis [24, 28].

Molecular docking suggested that Yinyanghuo C was stably bound to PTGS2, while it combined to both HK2 and GSK3B. In addition, MOL004384 of YYH binds GSK3B with the most affinity (−9.4 kcal/mol), MOL000358 (−9.3 kcal/mol), MOL012377 (−9.2 kcal/mol), MOL012372 (−9.1 kcal/mol) of MBZ, suggesting that, for GSK3B targets, these four active ingredients are likely to bind to it and regulate RA progression. It is noteworthy that the effect of MBZ on HK2 is mainly through MOL012372, MOL012375, MOL012377, MOL000263, and MOL000358 (all ≤−8 kcal/mol), while YYH mainly acts on HK2 through MOL003542, MOL004382, and MOL004384 (all ≤−9.5 kcal/mol). In addition, MOL012372 (−8.8 kcal/mol) and MOL000263 (−8.7 kcal/mol) of MBZ had the most affinity for binding to CASP3, followed by MOL004382 (−8.6 kcal/mol) of YYH. The sequence of binding to CASP8 was MOL000263 (−8.9 kcal/mol) in MBZ, followed by MOL004382 (−8.8 kcal/mol) in YYH and MOL012377 (−8.8 kcal/mol) in MBZ.

The glycogen synthase kinase 3b (GSK3b), a negative regulator of glucose homeostasis, is involved in energy metabolism, inflammation, and apoptosis pathways in a cell-type and environment-dependent manner. Active GSK3b is associated with mitochondrial quiescence in Drosophila oocytes. However, B cells deficient in GSK3B showed higher metabolic activity and stronger proliferation [29]. The current study identified GSK3b inactivation as a master regulator of macrophage metabolic reprogramming in RA synovial tissue [30, 31]. In RA, the regulatory role of GSK3b in preventing mitochondrial hyperactivity is lost, and GSK3B-Ser9 localizes to the mitochondria and stabilizes calcium transfer at mitochondrial associated membrane contact sites, a necessary mechanism to increase ATP production and drive tissue destructive effector functions [32]. While caspase-8 functions in synovial antigen-presenting cells to regulate the response to inflammatory stimuli by controlling the action of RIPK3, this delicate balance maintains joint homeostasis [33]. Active caspase 3 expression was increased in monocytes and synovial macrophages from RA patients compared with cells from healthy controls, and the use of caspase 3 inhibitors significantly blocked TNF-induced pyroptosis, which effectively alleviated arthritis in the CIA mouse model [34].

In summary, the MBZ active ingredients were predicted to mainly cause apoptosis and act as a major actor, while the YYH active ingredients acted as a regulator of glucose metabolism and hexokinase activity, playing an adjuvant role in RA. This provides a theoretical basis for the application of MBZ combined with YYH in the clinical treatment of RA and excavates new therapeutic drugs for treatment of RA. However, it is undeniable that there are still some shortcomings in this study. Network pharmacology relies too much on the existing database, and the composition and target prediction of TCM such as MBZ may be lagging behind and the data are not comprehensive. In addition, for the drug interactions, this study only made some predictions and inferences about the indirect effects between them. For the other targets of different components, the interaction mechanism was not predicted due to its extensive involvement. Our next work will provide more empirical findings and evidence.

## 5. Conclusion

In summary, MBZ and YYH act on RA through the interaction and coordination of different components. It may regulate the response of cells to drugs and the hypoxia environment by targeting GSK3b, HK2, caspase 3, and caspase 8 through the IL-17 signaling pathway and the TNF signaling pathway and inhibit the proliferation and glycolysis of RA FLS cells. Hence, it has a synergistic and attenuated therapeutic effect on RA.

## Figures and Tables

**Table 1:** Comparative analysis table of the two herbs.

Parameter	Momordicae semen	Epimedii herba
MW	347.4	414.72
AlogP	7.02	2.63
Hdon	1.23	3.86
Hacc	2.03	7.42
OB (%)	27.99	26.08
DL	0.39	0.37

**Table 2:** Information table of active ingredients of two herbs.

Mol ID	Molecule name	MW	AlogP	Hdon	Hacc	OB (%)	DL
*Momordicae semen*							
MOL012377	Bessisterol	412.77	7.64	1	1	42.98	0.76
MOL012375	Monachosorin A	404.54	4.76	2	4	37.3	0.71
MOL012374	Momordic acid	470.76	5.46	2	4	36.36	0.75
MOL006756	Schottenol	414.79	8.08	1	1	37.42	0.75
MOL006353	Stigmast-4-ene-3,6a-diol	430.79	7.12	2	2	34.37	0.78
MOL001297	trans-Gondoic acid	310.58	7.75	1	2	30.7	0.2
MOL000358	Beta-sitosterol	414.79	8.08	1	1	36.91	0.75
MOL000263	Oleanolic acid	456.78	6.42	2	3	29.02	0.76
MOL001640	NON	172.3	3.63	1	2	26.74	0.03
MOL007186	Karounidiol	440.78	5.96	2	2	26.26	0.77
MOL012372	(3R,4aR,6aS,6bS,8aS,11R,12aR,14bS)-4,4,6a,6b,8a,11,14b-heptamethyl-11-methylol-1,2,3,4a,7,8,9,10,12,12a,13,14-dodecahydropicen-3-ol	440.78	5.96	2	2	28.3	0.77

*Epimedii herba*							
MOL001510	24-Epicampesterol	400.76	7.63	1	1	37.58	0.71
MOL001645	Linoleyl acetate	308.56	6.85	0	2	42.1	0.2
MOL001771	Poriferast-5-en-3beta-ol	414.79	8.08	1	1	36.91	0.75
MOL001792	DFV	256.27	2.57	2	4	32.76	0.18
MOL003044	Chryseriol	300.28	2.32	3	6	35.85	0.27
MOL003542	8-Isopentenyl-kaempferol	354.38	3.63	4	6	38.04	0.39
MOL000359	Sitosterol	414.79	8.08	1	1	36.91	0.75
MOL000422	Kaempferol	286.25	1.77	4	6	41.88	0.24
MOL004367	Olivil	376.44	1.68	4	7	62.23	0.41
MOL004373	Anhydroicaritin	368.41	3.88	3	6	45.41	0.44
MOL004380	C-Homoerythrinan, 1,6-didYYHydro-3,15,16-trimethoxy-, (3.beta.)-	329.48	2.89	0	4	39.14	0.49
MOL004382	Yinyanghuo A	420.49	4.2	3	6	56.96	0.77
MOL004384	Yinyanghuo C	336.36	3.39	2	5	45.67	0.5
MOL004386	Yinyanghuo E	352.36	3.12	3	6	51.63	0.55
MOL004388	6-Hydroxy-11,12-dimethoxy-2,2-dimethyl-1,8-dioxo-2,3,4,8-tetrahydro-1H-isochromeno[3,4-h]isoquinolin-2-ium	370.41	2.75	1	6	60.64	0.66
MOL004391	8-(3-Methylbut-2-enyl)-2-phenyl-chromone	290.38	4.99	0	2	48.54	0.25
MOL004394	Anhydroicaritin-3-O-alpha-L-rhamnoside	676.73	0.77	8	15	41.58	0.61
MOL004396	1,2-bis (4-Hydroxy-3-methoxyphenyl) propan-1,3-diol	320.37	1.69	4	6	52.31	0.22
MOL004425	Icariin	676.73	0.77	8	15	41.58	0.61
MOL004427	Icariside A7	462.49	1.16	5	10	31.91	0.86
MOL000006	Luteolin	286.25	2.07	4	6	36.16	0.25
MOL000622	Magnograndiolide	266.37	1.18	2	4	63.71	0.19
MOL000098	Quercetin	302.25	1.5	5	7	46.43	0.28

**Table 3:** Information table of core target degree.

No.	Targets	Symbols	Degrees
1	Transcription factor AP-1	JUN	22
2	Caspase-3	CASP3	21
3	Peroxisome proliferator-activated receptor gamma	PPARG	18
4	Prostaglandin G/H synthase 2	PTGS2	17
5	Glycogen synthase kinase-3 beta	GSK3B	14
6	Caspase-8	CASP8	14
7	Heme oxygenase 1	HMOX1	11
8	Intercellular adhesion molecule 1	ICAM1	10
9	Hexokinase-2	HK2	3

## Data Availability

The figures and tables used to support the findings of this study are included within the article, and the original data are available from the corresponding author upon request.
